# Immunologic response in treatment-naïve HIV-2-infected patients: the IeDEA West Africa cohort

**DOI:** 10.7448/IAS.19.1.20044

**Published:** 2016-02-08

**Authors:** Eric Balestre, Didier Koumavi Ekouevi, Boris Tchounga, Serge Paul Eholie, Eugène Messou, Adrien Sawadogo, Rodolphe Thiébaut, Margaret T May, Jonathan Ac Sterne, François Dabis, the International Epidemiological Database to Evaluate AIDS (IeDEA) West Africa Collaboration

**Affiliations:** 1 University Bordeaux, ISPED, Centre INSERM U1219-Epidemiologie-Biostatistique, F-33000 Bordeaux, France; 2 INSERM, ISPED, Centre INSERM U1219-Epidemiologie-Biostatistique, F-33000 Bordeaux, France; 3 Programme PAC-CI, Centre Hospitalier Universitaire de Treichville, Abidjan, Côte d'Ivoire; 4 Service de Maladies Infectieuses et Tropicales, Centre Hospitalier Universitaire de Treichville, Abidjan, Côte d'Ivoire; 5 Département de Dermatologie-Infectiologie de l’Unité de Formation et de Recherche des Sciences médicales, Université Félix Houphouet Boigny, Abidjan, Côte d'Ivoire; 6 Centre de Prise en charge de Recherche et de Formation, Hôpital Yopougon Attié, Abidjan, Côte d'Ivoire; 7 Institut Supérieur des Sciences de la santé, Université Polytechnique de Bobo-Dioulasso, Nasso, Burkina Faso; 8 School of Social and Community Medicine, University of Bristol, Canynge Hall, Bristol, United Kingdom

**Keywords:** HIV-2, immunological response, antiretroviral treatment, linear mixed models, West Africa

## Abstract

**Introduction:**

Response to antiretroviral therapy (ART) among individuals infected with HIV-2 is poorly described. We compared the immunological response among patients treated with three nucleoside reverse-transcriptase inhibitors (NRTIs) to boosted protease inhibitor (PI) and unboosted PI-based regimens in West Africa.

**Methods:**

This prospective cohort study enrolled treatment-naïve HIV-2-infected patients within the International Epidemiological Databases to Evaluate AIDS collaboration in West Africa. We used mixed models to compare the CD4 count response to treatment over 12 months between regimens.

**Results:**

Of 422 HIV-2-infected patients, 285 (67.5%) were treated with a boosted PI-based regimen, 104 (24.6%) with an unboosted PI-based regimen and 33 (7.8%) with three NRTIs. Treatment groups were comparable with regard to gender (54.5% female) and median age at ART initiation (45.3 years; interquartile range 38.3 to 51.8). Treatment groups differed by clinical stage (21.2%, 16.8% and 17.3% at CDC Stage C or World Health Organization Stage IV for the triple NRTI, boosted PI and unboosted PI groups, respectively, *p*=0.02), median length of follow-up (12.9, 17.7 and 44.0 months for the triple NRTI, the boosted PI and the unboosted PI groups, respectively, *p*<0.001) and baseline median CD4 count (192, 173 and 129 cells/µl in the triple NRTI, the boosted PI and the unboosted PI-based regimen groups, respectively, *p*=0.003). CD4 count recovery at 12 months was higher for patients treated with boosted PI-based regimens than those treated with three NRTIs or with unboosted PI-based regimens (191 cells/µl, 95% CI 142 to 241; 110 cells/µl, 95% CI 29 to 192; 133 cells/µl, 95% CI 80 to 186, respectively, *p*=0.004).

**Conclusions:**

In this observational study using African data, boosted PI-containing regimens had better immunological response compared to triple NRTI combinations and unboosted PI-based regimens at 12 months. A randomized clinical trial is still required to determine the best initial regimen for treating HIV-2 infected patients.

## Introduction

Between one and two million people are estimated to be living with HIV-2 infection in West Africa [1], the region that is the epicentre of the HIV-2 epidemic, with prevalence apparently decreasing over time [2–5]. Although there is limited experience in the management of HIV-2 infection worldwide, it is well known that HIV-2 is naturally resistant to the non-nucleoside reverse-transcriptase inhibitors (NNRTIs) [6,7] that have been part of the standard first-line antiretroviral therapy (ART) for the treatment of HIV-1 infection in low-income countries for the past decade. In the 2010 treatment guidelines [8], the World Health Organization (WHO) recommended treating patients living with HIV-2 in limited-resource countries with an ART regimen containing a ritonavir-boosted protease inhibitor (PI) plus two nucleoside reverse-transcriptase inhibitors (NRTIs). Triple NRTI regimens were recommended only in patients with CD4 counts >200 cells/mm^3^. In the 2013 guidelines [9], WHO recommendations proposed a regimen containing three NRTIs or a ritonavir-boosted PI plus two NRTIs. If a PI-based regimen is used, the preferred option for first-line PI is lopinavir. However, this recommendation is based on weak evidence according to WHO grading criteria [8–11]. Although PIs are active against HIV-2, they show varying degrees of activity due to natural protease polymorphisms. A study that compared the potency of different PIs against HIV-2 showed that lopinavir, saquinavir, tipranavir and darunavir were the most potent [10]. Another study reported that saquinavir, lopinavir and darunavir are potent inhibitors of HIV-2 isolates [12].

In the context of the large and still ongoing scale-up of ART in the West African region, HIV-2 infection causes specific operational, clinical and public health challenges [13]. First, the lack of routinely available rapid antibody tests to differentiate between HIV-1 and HIV-2 can lead to delayed diagnoses and thus to initiation of inappropriate and ineffective first-line ART prescriptions [14,15]. Indeed, a previous study of our group [15] showed that despite international recommendations 17% of HIV-2-infected patients were initially treated with inappropriate NNRTI-containing regimens, resulting in poor immunological response mainly due to late confirmation of HIV-2 infection [15]. Second, the lack of commercial viral load quantification assay [16] impacts on the monitoring of HIV-2 infected patients [13,17].

Owing to its low prevalence and its geographical restriction to West Africa, response to ART in HIV-2 infection is still poorly understood [4,18]. There have been no randomized trials investigating the response to ART regimens in HIV-2-infected patients [19]. Only a few observational cohort studies with limited sample size have provided information on HIV-2-infected patients on ART in Europe and in the United States [20–26], as well as in West Africa [13–15,27–31]. Moreover, limited data are available on the response to the different first-line regimens used in HIV-2 patients.

Whereas the data have generally favoured boosted PI regimens over those comprised of triple NRTIs, to our knowledge, only one report from a European collaboration [20] and two from West Africa [15,30] explicitly compared the two first-line regimens. Using data from the International Epidemiological Database to Evaluate AIDS West Africa (IeDEA-WA) HIV-2 collaboration [32], we aimed to investigate the impact of first-line ART regimens on immunological response within the first 12 months of ART, for HIV-2 infected patients in the West Africa region where there are limited options for second-line regimens.

## Methods

### Description of the cohort

Since 2006 a network of adult and paediatric HIV clinics in West Africa has existed as part of the global IeDEA Collaboration (www.iedea.org/), funded by the US National Institutes of Health [32]. The IeDEA-WA HIV-2 cohort was recently established in order to better understand the epidemiology, care patterns and treatment of HIV-2 infection [33]. This cohort includes HIV-2 and dually HIV-1- and HIV-2-infected patients, on ART or not, followed in 12 clinics located in five West African countries (six in Côte d'Ivoire, two in Mali, two in Burkina Faso, one in Benin and one in Senegal).

### Data collection

Standardized questionnaires capturing the relevant information on HIV-2 care were developed with an electronic database implemented at the site level. All sites completed the specific questionnaires retrospectively and then prospectively and entered the data into the IeDEA-WA HIV-2 database. The databases from each site are sent every six months to the Regional Centre in Abidjan, Côte d'Ivoire, and Bordeaux, France, using compression/encryption software. Data collected include the following: 1) Baseline demographics and clinical data: birth date, gender, HIV clinical stage (WHO or CDC stage), ART initiated, clinical assessment, medical history; 2) Follow-up: clinical assessment (tuberculosis, other diseases/infection, HIV clinical stage, weight, height, medications such as antiretroviral drugs and co-trimoxazole); 3) Biological data: CD4 count, haemoglobin, aspartate transaminase, alanine transaminase and plasma HIV RNA viral load (when available); 4) Outcomes: death, loss to follow-up and transferred out.

### Inclusion criteria

Patients aged >17 years at ART initiation and with an HIV-2 infection confirmed by two or three rapid HIV tests based on country-specific national algorithms were eligible for this analysis. Only those patients who initiated ART with three NRTIs or a PI-based regimen were included. Patients without documented CD4 count at ART initiation and/or with unknown gender were excluded.

### Antiretroviral treatment

ART was provided to the HIV-2 infected patients according to the national guidelines. In West Africa, HIV-2 treatment guidelines were based on WHO recommendations [34]. Up to 2013, the recommended first-line regimen contained three NRTIs (tenofovir (TDF)+lamivudine (3TC) or emtricitabine (FTC)+zidovudine (AZT) or AZT+3TC+abacavir (ABC)). Since 2013, a boosted PI-based regimen has been recommended, with lopinavir being the preferred option. In patients for whom boosting the PI is contraindicated or not tolerated, a triple NRTI regimen was recommended (AZT+3TC or FTC+TDF or ABC).

### Follow-up and CD4 count measurement

After initiation into care, patients were typically followed up every six months or were seen in between scheduled visits if illness occurred. T-CD4 lymphocyte counts were measured every six months. The absolute CD4+/CD8+ T-cell counts were performed using standard flow cytometry (FACScan, Becton Dickinson).

### Statistical analysis

CD4 count trajectories over the first 12 months of ART were modelled using linear mixed models (LMMs), with baseline defined as the date of ART initiation. We included all CD4 count measurements between baseline (or the first CD4 count recorded within a window of six months prior to baseline) and 24 months of follow-up (or six months later at the latest). In the main analysis, patients who switched initial treatment were right censored at the date of switching. We modelled the CD4 count changes over time [35] using fractional polynomials of one and two degrees with powers −2, −1, −0.5, 0 (natural log), 0.5, 1, 2 and 3. The best-fitting fractional polynomial was selected by comparing the deviance of different models and had powers 0.5 and 1. Random effects on the different fractional polynomial terms accounted for the correlation of repeated measurements within each subject. For degree-2 models unstructured and diagonal covariance matrices were compared. Models with an unstructured covariance matrix were selected, as their Akaike information criterion was smaller. Univariable LMMs were used to assess covariate effects, and a manual backwards selection method was used to select significant variables in a multivariable LMM. To confirm the adequacy of the model, residual homoscedasticity and normality were graphically checked.

To address possible informative dropout we performed a sensitivity analysis restricted to patients remaining in care beyond the 12 months of ART (patients deceased or lost to follow-up were excluded). Patients were defined as lost to follow-up if they were not known to have died, not known to have transferred out and not seen at the clinic at least once in the last six months prior to the closure date of the database [36].

In a second sensitivity analysis, we performed the multivariable LMM in an intent-to-treat analysis.

## Results

### Selection of the study sample

**Figure 1:**
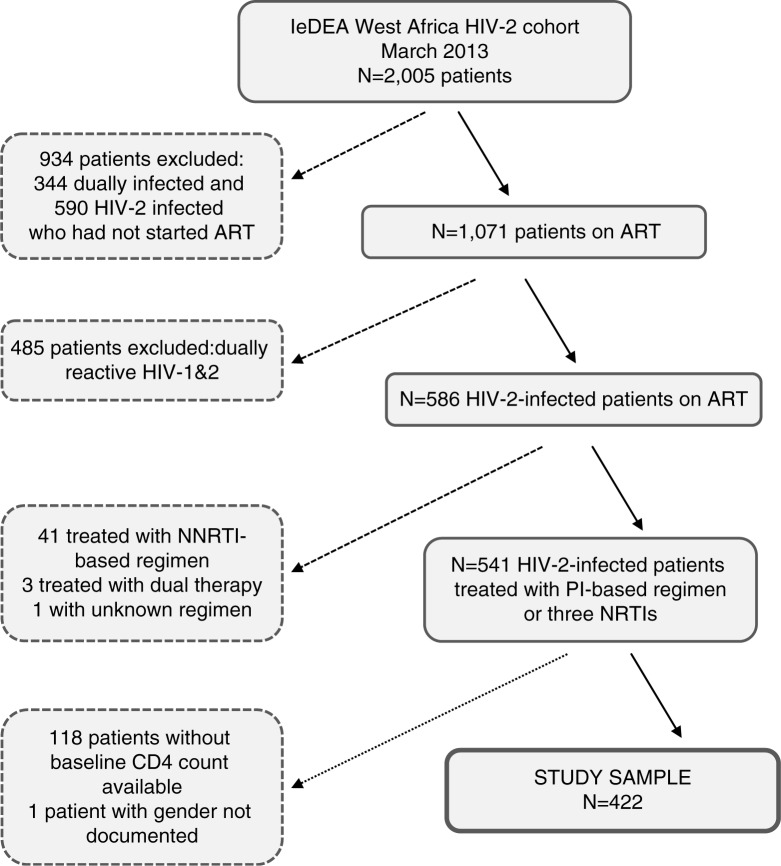
Flow chart of the selection of the study sample of patients infected only with HIV-2 and treated with a recommended antiretroviral treatment regimen, IeDEA West Africa Collaboration. ART, antiretroviral treatment; NNRTI, non-nucleoside reverse-transcriptase inhibitor; PI, protease inhibitor; NRTI, nucleoside reverse-transcriptase inhibitors

As of March 2013 the IeDEA-WA database contained 2005 patients infected with HIV-2 (or dually infected with HIV-1 and HIV-2). Figure 1 shows the different steps of the selection of the study sample. We excluded 590 HIV-2 patients and 344 dually infected patients because they were not treated with ART. We excluded 485 patients because they were dually infected, one patient with an unknown first-line ART regimen and 44 (7.5%) HIV-2 patients treated with a non-recommended first-line ART regimen. Among these 44 patients, 41 were initially treated with an NNRTI-based regimen and three with only two drugs. Of these 44 patients, 31 (70.5%) treated with a non-recommended ART regimen had a subsequent drug combination switch reported (28 (90.3%) switched to a PI-based regimen and three patients (9.7%) switched to a triple NRTI regimen), at a median 14 months after ART initiation (interquartile range (IQR) 2.7 to 21.3 months). After exclusion of 118 patients without documented baseline CD4 count measurements, there remained 422 eligible HIV-2-infected patients that initiated ART between 1999 and 2012 who constituted the core sample for this analysis.

### Sample characteristics

Of 422 eligible patients, 285 (67.5%) started a boosted PI-based ART regimen, 104 (24.6%) an unboosted PI-based regimen and 33 (7.8%) a triple NRTI regimen (Table 1). The subjects in the three groups had similar sex and age distributions, mortality and loss to follow-up, but varied markedly in their baseline clinical stage, baseline CD4 count, follow-up duration and the calendar year of starting ART (Table 1). Of the patients treated with a boosted or unboosted PI-based regimen, 17% had an advanced clinical stage (WHO IV or CDC C) compared to 21.2% of patients treated with three NRTIs (*p*=0.02). Baseline CD4 cell count was lower in the unboosted PI group (median 129 cells/µl; 67 to 206) compared to the boosted PI group (173 cells/µl; 80 to 265) and to the NRTI group (192 cells/µl; 114 to 308; *p*=0.003). Median follow-up time was higher for the unboosted PI group (median 44 months; 13.2 to 72.0) compared to 17.7 months (2.4 to 36.6) and 12.9 months (0 to 38.9) for patients treated with a boosted PI-based and a three-NRTI regimen, respectively (*p*<0.001). Twenty-one (63.6%) patients initially treated with three NRTIs started their first-line ART regimen between 2008 and 2012, compared to 191 (67.0%) patients treated with a boosted PI-based regimen and 9 (8.6%) patients treated with an unboosted PI-based regimen (*p*<0.001). Of the patients treated with a PI-based regimen (boosted or not) 55% were female, compared to 42.4% of the patients treated with three NRTIs (*p*=0.35). The median age at ART initiation was 45.5 (38.9 to 52.1), 44.2 (36.9 to 51.2) and 46.1 (40.7 to 51.9) years for patients treated with a boosted PI-based, unboosted PI-based and triple NRTI regimen, respectively (*p*=0.59). Twelve months after starting ART there were 12 (2.8%) deaths in total, with similar proportions in the three treatment groups (3.2%, 1.9% and 3.0% in the boosted PI, unboosted PI and NRTI groups, respectively; *p*=0.80). Twenty-four percent of patients were lost to follow-up after 12 months (*p*=0.43).

**Table 1:** Baseline characteristics of the HIV-2-infected patients on antiretroviral therapy (ART), IeDEA West Africa Collaboration (*n*=422)

	Initial ART regimen			
		
	Unboosted PI-based *n*=104	Boosted PI-based *n*=285	Three NRTIs *n*=33	*p*^a^
Country (%)				0.003
Côte d'Ivoire	71 (25.8)	177 (64.4)	27 (9.8)	
Burkina Faso	13 (15.8)	69 (84.2)	–	
Mali	12 (35.3)	21 (61.8)	1 (2.9)	
Senegal	8 (27.6)	16 (55.2)	5 (17.2)	
Benin	–	2 (100)	–	
Female (%)	58 (55.8)	158 (55.4)	14 (42.4)	0.348
Age (in years) median (IQR)	44.2 (36.9;51.2)	45.5 (38.9;52.1)	46.1 (40.7;51.9)	0.586
Baseline clinical stage (%)				0.020
WHO I/II or CDC A	12 (11.5)	64 (22.5)	4 (12.1)	
WHO III or CDC B	63 (60.6)	128 (44.9)	13 (39.4)	
WHO IV or AIDS	18 (17.3)	48 (16.8)	7 (21.2)	
missing	11 (10.6)	45 (15.8)	9 (27.3)	
Baseline haemoglobin (g/dl) median (IQR)	10.6 (9.6;11.9)	11.3 (9.8;12.5)	11.5 (10.2;12.3)	0.225
Baseline CD4 count (cells/µl) median (IQR)	129 (67;206)	173 (80;265)	192 (114;308)	0.003
Baseline CD4 count (%)				0.047
0 to 49	20 (19.2)	42 (14.7)	1 (3.0)	
50 to 99	21 (20.2)	44 (15.4)	5 (15.2)	
100 to 199	36 (34.6)	74 (26.0)	11 (33.3)	
200 to 349	21 (20.2)	85 (29.8)	10 (30.3)	
>349	6 (5.8)	40 (14.0)	6 (18.2)	
Year of ART initiation (%)				<0.001
<2004	17 (16.4)	6 (2.1)	9 (27.3)	
2004 to 2005	49 (47.1)	22 (7.7)	3 (9.1)	
2006 to 2007	29 (27.9)	66 (23.2)	–	
2008 to 2009	7 (6.7)	100 (35.1)	11 (33.3)	
2010 to 2012	2 (1.9)	91 (31.9)	10 (30.3)	
Follow-up duration (months) median (IQR)	44.0 (13.2;72.0)	17.7 (2.4;36.6)	12.9 (0;38.9)	<0.001
Deceased (%)^b^	2 (1.9)	9 (3.2)	1 (3.0)	0.800^c^
Lost to follow-up (%)^b^	20 (19.2)	73 (25.6)	8 (24.2)	0.426

IQR, interquartile range; PI, protease inhibitor; NRTI, nucleoside reverse-transcriptase inhibitor; WHO, World Health Organization

a chi-square test for qualitative variables and Kruskal-Wallis test for quantitative variables

b during the first 12 months after ART initiation

c Fisher's exact test.

The triple NRTI ART combinations prescribed were AZT+3TC and ABC for 22 patients (66.7%), stavudine+3TC and didanosine for six patients (18.2%), stavudine+3TC and ABC for three patients (9.1%) and AZT+3TC and didanosine for two patients (6.1%). For patients treated with a boosted PI-based regimen, the initial combinations mostly prescribed were AZT+3TC and lopinavir-ritonavir for 130 patients (45.6%), stavudine+3TC and lopinavir-ritonavir for 52 patients (18.3%) and AZT+3TC+indinavir and ritonavir for 34 patients (11.9%). For patients treated with an unboosted PI-based regimen, the initial combinations mostly prescribed were AZT+3TC+indinavir for 61 patients (58.7%), stavudine+3TC+indinavir for 25 patients (24.0%) and AZT+3TC+nelfinavir for 9 patients (8.7%). Lopinavir-ritonavir was the most frequently prescribed PI for 73.7% in the boosted PI group (*n*=210), followed by indinavir for 25.3% (*n*=72) and saquinavir for three patients (1.1%). In the unboosted PI group 88 (84.6%) patients were treated with indinavir and 16 (15.4%) with nelfinavir.

Treatment modification during the first 12 months of ART was reported for 11 (33.3%) patients initially treated with three NRTIs: five patients switched to a boosted PI-based regimen, four to another NRTI-based combination and two to an NNRTI-based regimen. Of 45 (15.8%) patients initially treated with a boosted PI-based regimen who had a treatment modification within 12 months, 38 were still treated with a boosted PI-based regimen, three were treated with three NRTIs, two with a NNRTI combination, one with an unboosted PI-based regimen and one with two NRTIs. Of 27 (26.0%) patients initially treated with an unboosted PI-based regimen, 24 were treated with a boosted PI-based regimen, two with an NNRTI combination and one with three NRTIs. The median times from ART initiation to treatment modification were 5.7 (2.9 to 8.9), 4.9 (2.8 to 8.7) and 5.8 (2.0 to 8.8) months for patients initially treated with three-NRTI, unboosted PI-based and boosted PI-based regimens, respectively. Reasons for ART modification were not recorded in the database.

### Immunologic response to ART treatment

**Figure 2:**
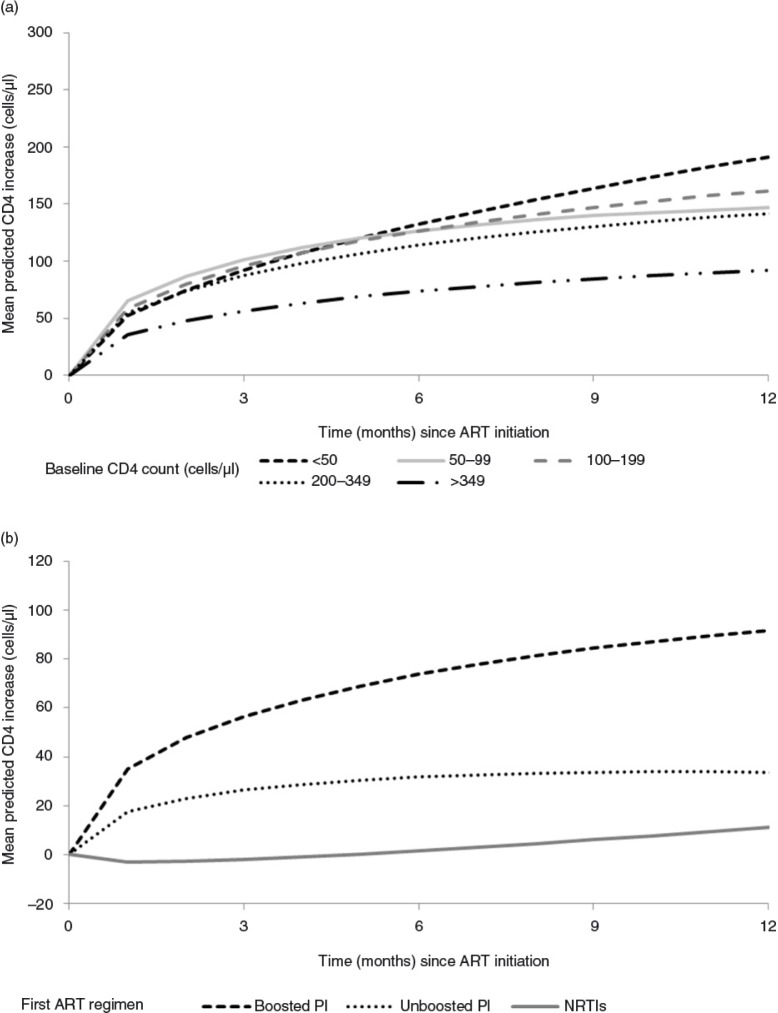
Mean adjusted CD4 count change after antiretroviral treatment initiation according to baseline CD4 count (2a – top panel) and by antiretroviral treatment regimen (2b – bottom panel), IeDEA West Africa Collaboration.

Figure 2a shows trajectories of CD4 count recovery estimated by the multivariable LMM stratified by CD4 count category at baseline. In the reference group (women treated with a boosted PI-based regimen and with an initial CD4 count <50 cells/µl), the mean CD4 changes were 132 cells/µl (95% CI 89 to 176) and 191 cells/µl (95% CI 142 to 241) at 6 and 12 months after starting ART, respectively. Table 2 shows estimated mean CD4 changes at 6 and 12 months after starting ART. The CD4 count response to ART within the first 12 months of ART was associated with the absolute value of the baseline CD4 count (*p*=0.0291), with gender (*p*=0.0302) and with the type of first ART regimen (*p*=0.0045). Compared to patients with a baseline CD4 count <50 cells/µl, patients with baseline CD4 count ≥350 had lower CD4 count recovery (−99 cells/µl (−164 to −34)) 12 months after ART initiation. Compared to women, men had a lower CD4 count recovery after 12 months of ART (−39 cells/µl (−71 to −8)). We found no association between CD4 response to ART and age, baseline haemoglobinaemia, year of ART initiation, country and initial clinical stage (data not shown).

**Table 2:** Mean CD4 count changes at 6 and 12 months compared to the reference group^a^ and estimated with multivariable linear mixed model (*N*=422; 1341 observations), IeDEA West Africa Collaboration

Variables	Mean CD4 change difference (cells/µl) at 6 months (95% CI)	Mean CD4 change difference (cells/µl) at 12 months (95% CI)	*p*
Baseline CD4 count (cells/µl)			0.0291
<50	Reference^b^	Reference^c^	
50 to 99	−6 (−59 to +48)	−45 (−105 to +16)	
100 to 199	−6 (−53 to +41)	−29 (−83 to +24)	
200 to 349	−18 (−66 to +29)	−49 (−104 to +5)	
≥350	−59 (−116 to −1)	−99 (−164 to −34)	
Gender			0.0302
Female	Reference^b^	Reference^c^	
Male	−26 (−54 to +2)	−39 (−71 to −8)	
First ART regimen			0.0045
Boosted PI-based	Reference^b^	Reference^c^	
Unboosted PI-based	−42 (−74 to −10)	−58 (−94 to −23)	
NRTI-based	−72 (−129 to −16)	−81 (−148 to −14)	

a Females with initial CD4 count <50 cells/µl treated with boosted PI-based regimen; CI, confidence interval; ART, antiretroviral treatment; PI, protease inhibitor; NRTI, nucleoside reverse-transcriptase inhibitors

b the mean CD4 count change for the reference group at 6 months was 132 cells/µl (95% CI=89; 176)

c the mean CD4 count change for the reference group at 12 months was 191 cells/µl (95% CI=142; 241).

There was a strong association between the type of first-line ART regimen and the overall CD4 response trajectory (*p*=0.0045). Compared to patients initially treated with a boosted PI-based regimen, CD4 count recovery was lower for patients treated with three NRTIs or with an unboosted PI-based regimen (−72 cells/µl (−129 to −16); −42 cells/µl (−74 to −10), respectively) at six months. The difference in CD4 count recovery between the three treatment groups persisted at 12 months (Figure 2b).

**Figure 3:**
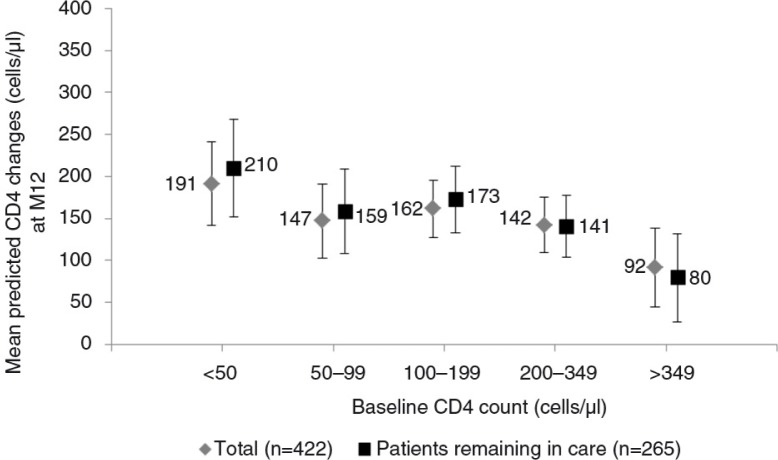
Adjusted mean CD4 count change at M12 (cells/µl) with 95% confidence interval, by baseline CD4 count (cells/µl) for the reference group (female treated by a first-line antiretroviral treatment regimen including a boosted protease inhibitor). Estimation by multivariable linear mixed model for patients remaining in care (*n*=265) and for all patients (*n*=422), IeDEA West Africa Collaboration.

The results of the sensitivity analysis restricted to patients remaining in care (*n*=265) were similar to those from the main analysis. CD4 count changes at 12 months estimated from these two models, according to baseline CD4 count, are shown in Figure 3.

The results of the second sensitivity intent-to-treat analysis were similar to those from the main analysis.

## Discussion

Based on a collaborative analysis of data combined from 12 clinics located in five West African countries, we described immunological responses of HIV-2 infected patients in the largest sample of HIV-2-infected patients followed for up to 12 months on ART. We compared the ART regimens recommended by the WHO for low- and middle-income settings and found CD4 count responses to be higher for patients treated with boosted PI-based regimens than for those treated with triple NRTI or unboosted PI-based regimens 6 and 12 months after ART initiation.

Another study that investigated the immunological responses to the two first-line ART regimens for HIV-2 infected patients, the European cohort study ACHI_E_V_2E_ [20], showed a lower immunological response for patients initially treated with three NRTIs than those treated with a PI-based regimen. Indeed, a CD4 count decrease was observed for patients treated with three NRTIs (−60 cells/µl per year) and a CD4 count increase for those treated with a PI-based regimen (+76 cells/µl per year). At 12 months, the CD4 counts for patients treated with three NRTIs and a PI-based regimen were 191 and 327 cells/µl, respectively. The immunological response beyond month 12 was not investigated in that study. Whether the early immunological advantage observed with boosted PI-based ART translates into clinical benefits cannot be answered here and will be difficult to investigate.

In light of the results of the European cohort study [20], in 2013 a French group of experts [37] recommended the use of a PI-based regimen as a first-line combination and ceased to recommend the use of a triple NRTI-based regimen for HIV-2 infected patients. The choice between the two first-line regimens is probably more critical in low-income countries: On the one hand, therapies based on a combination of three NRTIs are an interesting alternative in a context of high tuberculosis prevalence because treatment with a rifampin-based regimen requires double boosting of ritonavir in association with lopinavir or saquinavir, which results in an increased risk of hepatotoxicity [38]. On the other hand, the risk of occurrence of drug resistance has to be balanced against this as there are limited options for second-line regimens. We recently reported that, after a median duration of four years on PI-based regimens, 74% of 145 HIV-2-infected patients had suppressed viral loads of <50 copies/ml; however, HIV-2 resistance mutations to NRTIs and PIs were detected in 21 of 25 (84%) and 20 of 29 (69%) samples, respectively, despite adequate antiretroviral plasma concentrations [39]. This study clearly showed that in cases of virological failure there is a limited HIV-2 therapeutic arsenal and that cross-resistance dramatically reduced second-line treatment options. Another study reported that 40% of patients with PI resistance mutations appeared to be resistant to darunavir, which is recommended as second-line therapy for HIV-2 patients [40]. With regard to the HIV-2 therapeutic arsenal, in cases of NRTI and PI resistance, the only active drug class is integrase inhibitors and possibly the CCR5 inhibitor maraviroc. However, the use of integrase inhibitors may be limited in pretreated patients who require a combination with fully active drugs and who harbour NRTI-resistant viruses because of the low genetic barrier to resistance of this drug class. The potential use of integrase inhibitors and maraviroc is also limited by the high costs of these medicines.

Among patients who initiated triple NRTI regimens, one-third had changed treatment over the period of 12 months. This rate was higher compared to other African public health ART programmes treating HIV-1 patients and needs further exploration to understand the reasons for treatment modification among HIV-2 patients. The most obvious explanation for the high switch rate is the known inferiority of triple NRTI regimens and co-treatment of tuberculosis being the main reason for their use. Side effects, tolerability or adherence could not be investigated as they were not available in our database. In the European HIV-2 cohort study, treatment modification was less common with switching reported in 12% of patients in the PI group and 18% in the NRTI group [20].

A limitation of our study was the small number and limited follow-up duration of HIV-2 infected patients treated with three NRTIs (17 of the 33 patients had at least one CD4 count documented after six months). Indeed, in the WHO guidelines of 2010 [8] this ART regimen was only recommended for patients with a CD4 cell count >200 cells/µl. In the 2013 WHO consolidated guidelines [9] this eligibility criteria has been dropped, which could lead to triple NRTI regimens being prescribed more widely.

Another limitation is the use of suboptimal triple NRTI regimens in West Africa. Some combinations used in this study, such as AZT+3TC+ABC, are not well tolerated and are known to be less potent than triple NRTI regimens used elsewhere in Africa.

Because the three groups of patients compared in our study differed in terms of baseline CD4 count, baseline clinical stage and follow-up duration, this analysis was possibly affected by selection bias and the results should be interpreted with caution, as in all observational cohorts comparing treatment regimens [41]. In resource-limited countries, high rates of patients lost to follow-up are frequently observed [42,43], which could induce informative dropout [44]. However the results of the sensitivity analysis we conducted on patients remaining in care were similar to those of the main analysis, showing that this informative dropout bias was limited.

In conclusion this study further demonstrates the inferiority of unboosted indinavir and triple nucleoside regimens for the treatment of HIV-2 infection. Limitations inherent in this observational design will be addressed by the First-Line Treatment for HIV-2 trial (FIT-2, NCT02150993) already underway in five countries in West Africa and funded by the *Agence Nationale de Recherches sur le Sida et les hépatites virales* (ANRS). The critical issue of second-line regimens for HIV-2, including the optimal sequence of integrase inhibitors and/or boosted PIs, could potentially be addressed by a study enrolling the subjects failing therapy in those two arms of the FIT-2 trial.

## Appendix

The International Epidemiological Database to Evaluate AIDS – West Africa Collaboration Study Group (as of January 2014)

Participating sites (*members of the Steering Committee, ^§^members of the Executive Committee)

**Cotonou, Benin**

*Adults*: Djimon Marcel Zannou*, Carin Ahouada, Jocelyn Akakpo, Christelle Ahomadegbé, Jules Bashi, Alice Gougounon-Houéto, Angèle Azon-Kouanou, Fabien Houngbé, Jean Sehonou (CNHU Hubert Maga).

*Paediatrics*: Sikiratou Koumakpaï*^§^, Florence Alihonou, Marcelline d'Almeida, Irvine Hodonou, Ghislaine Hounhoui, Gracien Sagbo, Leïla Tossa-Bagnan, Herman Adjide (CNHU Hubert Maga).

**Burkina Faso**

*Adults*: Joseph Drabo*, René Bognounou, Arnaud Dienderé, Eliezer Traore, Lassane Zoungrana, Béatrice Zerbo (CHU Yalgado, ***Ouagadougou***), Adrien Bruno Sawadogo*^§^, Jacques Zoungrana, Arsène Héma, Ibrahim Soré, Guillaume Bado, Achille Tapsoba (CHU Souro Sanou, ***Bobo-Dioulasso***)

*Paediatrics*: Diarra Yé*, Fla Kouéta, Sylvie Ouedraogo, Rasmata Ouédraogo, William Hiembo, Mady Gansonré (CH Charles de Gaulle, ***Ouagadougou***).

**Abidjan, Côte d'Ivoire**

*Adults*: Eugène Messou*, Joachim Charles Gnokoro, Mamadou Koné, Guillaume Martial Kouakou, (ACONDA-CePReF); Clarisse Amani Bosse*, Kouakou Brou, Achi Isidore Assi (ACONDA-MTCT-Plus); Henri Chenal*, Denise Hawerlander, Franck Soppi (CIRBA); Albert Minga*, Yao Abo, Jean-Michel Yoboue (CMSDS/CNTS); Serge Paul Eholié*^§^, Mensah Deborah Noelly Amego, Viviane Andavi, Zelica Diallo, Frédéric Ello, Aristophane Koffi Tanon (SMIT, CHU de Treichville), Serge Olivier Koule*, Koffi Charles Anzan, Calixte Guehi (USAC, CHU de Treichville).

*Paediatrics*: Edmond Addi Aka*, Koffi Ladji Issouf, Jean-Claude Kouakou, Marie-Sylvie N'Gbeche, (ACONDA-CePReF); Touré Pety*, Divine Avit-Edi (ACONDA-MTCT-Plus); Kouadio Kouakou*, Magloire Moh, Valérie Andoblé Yao (CIRBA); Madeleine Amorissani Folquet*, Marie-Evelyne Dainguy, Cyrille Kouakou, Véronique Tanoh Méa-Assande, Gladys Oka-Berete, Nathalie Zobo, Patrick Acquah, Marie-Berthe Kokora (CHU Cocody); Tanoh François Eboua*, Marguerite Timité-Konan, Lucrèce Diecket Ahoussou, Julie Kebé Assouan, Mabéa Flora Sami, Clémence Kouadio (CHU Yopougon).

**Accra, Ghana**

*Paediatrics*: Lorna Renner*^§^, Bamenla Goka, Jennifer Welbeck, Adziri Sackey, Seth Ntiri Owiafe (Korle Bu TH).

**Guinea-Bissau**

*Adults*: Christian Wejse*^§^, Zacarias José Da Silva*, Joao Paulo (Bandim Health Project); The Bissau HIV cohort study group: Amabelia Rodrigues (Bandim Health Project), David da Silva (National HIV Programme Bissau), Candida Medina (Hospital National Simao Mendes, Bissau), Ines Oliviera-Souto (Bandim Health Project), Lars Østergaard (Department of Infectious Diseases, Aarhus University Hospital), Alex Laursen (Department of Infectious Diseases, Aarhus University Hospital), Morten Sodemann (Department of Infectious Diseases, Odense University Hospital), Peter Aaby (Bandim Health Project), Anders Fomsgaard (Department of Virology, Statens Serum Institut, Copenhagen), Christian Erikstrup (Department of Clinical Immunology), Jesper Eugen-Olsen (Department of Infectious Diseases, Hvidovre Hospital, Copenhagen).

**Bamako, Mali**

*Adults*: Moussa Y Maïga*^§^, Fatoumata Fofana Diakité, Abdoulaye Kalle, Drissa Katile (CH Gabriel Toure), Hamar Alassane Traore*, Daouda Minta*, Tidiani Cissé, Mamadou Dembelé, Mohammed Doumbia, Mahamadou Fomba, Assétou Soukho Kaya, Abdoulaye M Traoré, Hamady Traoré, Amadou Abathina Toure (CH Point G).

*Paediatrics*: Fatoumata Dicko*, Mariam Sylla, Alima Berthé, Hadizatou Coulibaly Traoré, Anta Koïta, Niaboula Koné, Clémentine N'Diaye, Safiatou Touré Coulibaly, Mamadou Traoré, Naïchata Traoré (CH Gabriel Toure).

**Nigeria**

*Adults*: Man Charurat* (UMB/IHV), Vivian Kwaghe*, Samuel Ajayi, Georgina Alim, Stephen Dapiap, Otu (UATH, ***Abuja***), Festus Igbinoba (National Hospital ***Abuja***), Okwara Benson*, Clément Adebamowo*, Jesse James, Obaseki, Philip Osakede (UBTH, ***BeninCity***), John Olasode (OATH, ***Ile-Ife***).

**Dakar, Senegal**

*Adults*: Moussa Seydi*, Papa Salif Sow, Bernard Diop, Noël Magloire Manga, Judicael Malick Tine^§^, Coumba Cissé Bassabi (SMIT, CHU Fann).

*Paediatrics*: Haby Signate Sy*, Abou Ba, Aida Diagne, Hélène Dior, Malick Faye, Ramatoulaye Diagne Gueye, Aminata Diack Mbaye (CH Albert Royer).

**Lomé, Togo**

*Adults*: Akessiwe Patassi*, Awèrou Kotosso, Benjamin Goilibe Kariyare, Gafarou Gbadamassi, Agbo Komi, Kankoé Edem Mensah-Zukong, Pinuwe Pakpame (CHU Tokoin/Sylvanus Olympio).

*Paediatrics*: Koko Lawson-Evi*^§^, Yawo Atakouma, Elom Takassi, Améyo Djeha, Ayoko Ephoévi-gah, Sherifa El-Hadj Djibril (CHU Tokoin/Sylvanus Olympio).

**Executive Committee*:** François Dabis (Principal Investigator, Bordeaux, France), Emmanuel Bissagnene (Co-Principal Investigator, Abidjan, Côte d'Ivoire), Elise Arrivé (Bordeaux, France), Patrick Coffie (Abidjan, Côte d'Ivoire), Didier Ekouevi (Abidjan, Côte d'Ivoire), Antoine Jaquet (Bordeaux, France), Valériane Leroy (Bordeaux, France), Charlotte Lewden (Bordeaux, France), Annie J Sasco (Bordeaux, France).

**Operational and Statistical Team:** Jean-Claude Azani (Abidjan, Côte d'Ivoire), Eric Balestre (Bordeaux, France), Serge Bessekon (Abidjan, Côte d'Ivoire), Sophie Karcher (Bordeaux, France), Jules Mahan Gonsan (Abidjan, Côte d'Ivoire), Jérôme Le Carrou (Bordeaux, France), Séverin Lenaud (Abidjan, Côte d'Ivoire), Célestin Nchot (Abidjan, Côte d'Ivoire), Karen Malateste (Bordeaux, France), Amon Roseamonde Yao (Abidjan, Côte d'Ivoire).

**Administrative Team:** Abdoulaye Cissé (Abidjan, Côte d'Ivoire), Alexandra Doring^§^ (Bordeaux, France), Adrienne Kouakou (Abidjan, Côte d'Ivoire), Guy Gneppa (Abidjan, Côte d'Ivoire), Elodie Rabourdin (Bordeaux, France), Jean Rivenc (Pessac, France).

**Consultants/Working Groups:** Xavier Anglaret (Bordeaux, France), Boubacar Ba (Bamako, Mali), Renaud Becquet (Bordeaux, France), Juan Burgos Soto (Bordeaux, France), Jean Bosco Essanin (Abidjan), Andrea Ciaranello (Boston, MA, USA), Sébastien Datté (Abidjan, Côte d'Ivoire), Sophie Desmonde (Bordeaux, France), Jean-Serge Elvis Diby (Abidjan, Côte d'Ivoire), Geoffrey S.Gottlieb* (Seattle, WA, USA), Apollinaire Gninlgninrin Horo (Abidjan, Côte d'Ivoire), Julie Jesson (Bordeaux, France), Serge N'zoré Kangah (Abidjan, Côte d'Ivoire), David Meless (Abidjan, Côte d'Ivoire), Aida Mounkaila-Harouna (Bordeaux, France), Camille Ndondoki (Bordeaux, France), Caroline Shiboski (San Francisco, CA, USA), Boris Tchounga (Abidjan, Côte d'Ivoire), Rodolphe Thiébaut (Bordeaux, France), Gilles Wandeler (Dakar, Senegal).

**Funding:** The National Cancer Institute, the Eunice Kennedy Shriver National Institute of Child Health and Human Development and the National Institute of Allergy and Infectious Diseases of the US National Institutes of Health, as part of the International Epidemiologic Databases to Evaluate AIDS (IeDEA) under award number U01AI069919. The content is solely the responsibility of the authors and does not necessarily represent the official views of the National Institutes of Health.

**Coordinating Centre:** ISPED, Univ Bordeaux Segalen, Bordeaux, France

**Regional Office:** PAC-CI, Abidjan, Côte d'Ivoire

**Methodologic Support:** MEREVA, Bordeaux, France

**Website:** http://www.mereva.net/iedea
